# Mesenchymal stem cells alleviate Japanese encephalitis virus-induced neuroinflammation and mortality

**DOI:** 10.1186/s13287-017-0486-5

**Published:** 2017-02-16

**Authors:** Peiyu Bian, Chuantao Ye, Xuyang Zheng, Jing Yang, Wei Ye, Yuan Wang, Yun Zhou, Hongwei Ma, Peijun Han, Hai Zhang, Ying Zhang, Fanglin Zhang, Yingfeng Lei, Zhansheng Jia

**Affiliations:** 1 Department of Infectious Diseases, Tangdu Hospital, the Fourth Military Medical University, Xi’an, 710038 China; 2 Department of Microbiology, School of Preclinical Medicine, the Fourth Military Medical University, Xi’an, 710032 China; 3 Laboratory Animal Center, the Fourth Military Medical University, Xi’an, 710032 China

**Keywords:** Mesenchymal stem cells, Immunomodulation, Japanese encephalitis virus, Inflammation

## Abstract

**Background:**

Japanese encephalitis virus (JEV) is the leading cause of viral encephalitis in Asia. Japanese encephalitis (JE) caused by JEV is characterized by extensive inflammatory cytokine secretion, microglia activation, blood-brain barrier (BBB) breakdown, and neuronal death, all of which contribute to the vicious cycle of inflammatory damage. There are currently no effective treatments for JE. Mesenchymal stem cells (MSCs) have been demonstrated to have a therapeutic effect on many central nervous system (CNS) diseases by regulating inflammation and other mechanisms.

**Methods:**

In vivo, 8- to 10-week-old mice were infected intraperitoneally with JEV and syngeneic bone marrow MSCs were administered through the caudal vein at 1 and 3 days post-infection. The mortality, body weight, and behavior were monitored daily. Brains from each group were harvested at the indicated times for hematoxylin and eosin staining, immunohistochemical observation, flow cytometric analysis, TUNEL staining, Western blot, quantitative real-time polymerase chain reaction, and BBB permeability assays. In vitro, co-culture and mixed culture experiments of MSCs with either microglia or neurons were performed, and then the activation state of microglia and survival rate of neurons were tested 48 h post-infection.

**Results:**

MSC treatment reduced JEV-induced mortality and improved the recovery from JE in our mouse model. The inflammatory response, microglia activation, neuronal damage, BBB destruction, and viral load (VL) were significantly decreased in the MSC-treated group. In co-culture experiments, MSCs reprogrammed M1-to-M2 switching in microglia and improved neuron survival. Additionally, the VL was decreased in Neuro2a cells in the presence of MSCs accompanied by increased expression of interferon-α/β.

**Conclusion:**

MSC treatment alleviated JEV-induced inflammation and mortality in mice.

**Electronic supplementary material:**

The online version of this article (doi:10.1186/s13287-017-0486-5) contains supplementary material, which is available to authorized users.

## Background

Viral encephalitis caused by multiple emerging and re-emerging viruses is characterized by overwhelming inflammation in the brain [1]. There are currently no effective methods to eliminate the virus or to cure the neuroinflammation, leading to thousands of people succumbing to the devastating illness and the survivors often suffering from permanent neurological deficits. Japanese encephalitis (JE) caused by the JE virus (JEV) is the most prevalent type of viral encephalitis [2].

JEV is a single-stranded, positive-sense RNA (~11 kb, monopartite, linear) virus belonging to the genus *Flavivirus* in the family *Flaviviridae* [3]. Globally, more than 67,900 cases of JEV infection are reported annually, among which approximately 30% are fatal and 50% suffer from permanent neuropsychiatric sequelae [4, 5]. Children are more susceptible to JEV, but there is an increasing occurrence in the middle-aged population [6]. Although the development of a JE vaccine has markedly reduced the incidence, its protection is not always effective [7]. JE is characterized by extensive neuroinflammation in the central nervous system (CNS) with robust and uncontrolled production of pro-inflammatory cytokines (e.g., tumor necrosis factor (TNF)-α and interferon (IFN)-γ) and chemokines (e.g., MCP-1/CCL2) [8–11]. Increased activation of microglia following JEV infection also contributes to the inflammatory response. During JE, neurons can be damaged by JEV directly or indirectly by the cytokine storm through the bystander effect [12]. Meanwhile, breakdown of the blood-brain barrier (BBB) integrity also accelerates the progression of JE [13]. There are currently no effective anti-JEV or other satisfactory therapeutic methods but life-sustaining treatment [14].

Mesenchymal stem cells (MSCs) have been demonstrated to contribute to tissue regeneration and modulate inflammation since they were first discovered by Friedenstein in 1974 [15, 16]. MSCs have the capacity for immunoregulation, the potential to migrate to the injury site, and the ability to differentiate into multiple cell types such as adipocytes, osteocytes, chondrocytes, and neuron-like cells. All of these properties have been beneficial in treating a wide spectrum of diseases in clinical and basic studies [17–19]. It has been reported that transplantation of MSCs can upregulate the expression of brain-derived neurotrophic factor (BDNF) and nerve growth factor (NGF), and can improve neurological recovery in many CNS diseases [20–23]. MSCs also control local inflammation and maintain tissue homeostasis by enhancing the innate and adaptive immune responses as well as regulating the activation and function of microglia [24, 25]. In addition, MSCs regulate BBB integrity by promoting the expression of vascular endothelial growth factor and angiogenesis [26–28]. Thus, the functional features of MSCs indicate their great potential in JE treatment. However, it is unknown whether MSCs can attenuate the encephalitis caused by JEV.

This study is the first to show that MSC treatment reduced the mortality and neurological pathology in the mouse model of JE. We also demonstrated that the beneficial effect of MSCs was mediated by suppressing the overactivation of microglia, reducing neuronal death, and improving the integrity of the BBB. Furthermore, we found that MSCs decreased JEV replication through the expression of IFN-β and IFN-α.

## Methods

### Ethics statement

All the mice used in this experiment were purchased from the Laboratory Animal Center of the Fourth Military Medical University (FMMU). All animal experiments were approved by the Animal Care and Use Committee of the FMMU.

### Isolation and culturing of mouse bone marrow MSCs

The mouse bone marrow-derived MSCs were isolated by the adhesive screening method [29]. Female *BALB/c* mice (aged 4 to 6 weeks) were euthanized by cervical dislocation and sterilized with 75% alcohol for 3 to 5 min. The femurs were removed, and both ends of each femur were cut off. The marrow cavities were then flushed with low-glucose Dulbecco’s modified Eagle’s medium (L-DMEM; Gibco, Grand Island, NY, USA) containing 10% fetal bovine serum (FBS; Gibco, Grand Island, NY, USA) and 1% penicillin-streptomycin (Gibco, Grand Island, NY, USA) repeatedly until the femurs turned white. The cell suspension was gently pipetted to separate the cells, transferred to a 15-ml centrifuge tube, and centrifuged at 1000 rpm for 5 min. The cell pellets were then resuspended in 5 ml L-DMEM, plated in 25-cm^2^ flasks, and cultured at 37 °C in an atmosphere with 5% CO_2_ and saturated humidity. After 24 h, the medium was first changed, with subsequent changes occurring every 3 days to remove the nonadherent cells. Plastic-adherent cells were cultured to 95% confluency and passaged with trypsinization. MSCs used in this study were from passages 5-8th and analyzed by flow cytometry (defining criteria: >95% positive for the stem cell surface antigens CD44 and Sca-1, and <5% negative for CD45, CD34, and I-A/I-E) and multi-differentiation evaluation (adipocytes, osteocytes, and chondrocytes, using the Mouse Mesenchymal Stem Cell Functional Identification Kit (Cyagen, China)) before the experiments (see Additional file 1: Figure S1).

### Cells and virus

The JEV SA-14 strain was propagated in the mosquito cell line C6/36. The supernatant containing JEV was concentrated at 100:1 and stored in aliquots at –80 °C until further use. The virus stocks were titrated by a conventional plaque assay [30].

The microglia cell line N9 and the neuroblast cell line Neuro2a were purchased from ATCC and maintained in 5% FBS DMEM and 10% FBS DMEM, respectively.

### MSC transplantation into JEV-infected mice

Weight-matched adult female *BALB/c* mice (8–10 weeks old) were randomly divided into three groups: control group (phosphate-buffered saline (PBS)); JEV-infected group (JEV); and JEV-infected and MSC-treated group (JEV + MSC). The JEV and JEV + MSC mice were intraperitoneally injected with 5 × 10^7^ PFU of JEV in 200 μl PBS. For the cellular injections, 200 μl of either PBS alone or MSCs (5 × 10^5^ cells per mouse) were intravenously administered to mice belonging to the JEV and JEV + MSC groups, respectively, at 1 and 3 days post-infection (dpi). The mice in each group were monitored daily to assess behavior, weight, and mortality. Behavioral scoring was performed according to the criteria previously described with minor modifications [31], and the mean scores of all mice in each group were calculated. After the comparative observations were repeated three times, we found an obvious difference in the manifestation of the clinical syndrome between the JEV and JEV + MSC groups from 6 dpi. Subsequently, new models and treatments were conducted and brains were collected at 6 dpi for further experiments.

### Quantitative real-time polymerase chain reaction (qRT-PCR)

At 6 dpi, mice were anesthetized with 2% pentobarbital sodium (0.1 ml/10 g body weight) and perfused with 1× PBS, and then the whole brain of each mouse from all the groups was harvested and stored at –80 °C. Viral burden and the expression of cytokines and chemokines were determined by relative qRT-PCR (see Additional file 2: Table S1 for the primer sequences). Total RNA from each whole brain and the in vitro cultured cells was extracted with RNAfast1000 (PIONEER, China). The first-strand cDNA was prepared by reverse transcription with total RNA as the template using a PrimeScript RT reagent Kit (TaKaRa, Japan). All qRT-PCR experiments were performed using SYBR Green Real-Time PCR Master Mix (TaKaRa, Japan) according to the manufacturer’s instructions.

### Hematoxylin and eosin (H&E) and immunohistochemical (IHC) staining

At 6, 10, and 22 dpi, mice were anesthetized with 2% pentobarbital sodium (0.1 ml/10 g body weight) and perfused with 1× PBS followed by 4% paraformaldehyde (PFA) for 30 min. The brain was removed, fixed in 4% PFA for 12 h, and then cryoprotected in 30% sucrose. Tissue sections of 10 μm thickness were prepared with a vibratome. The standard H&E staining protocol was followed for tissue staining. For IHC staining, primary antibodies were incubated at room temperature for 16 h. Corresponding secondary antibodies were then incubated for 4 h at room temperature protected from light (see Additional file 3: Table S2 for the antibodies). The nuclei were counterstained with DAPI (100 ng/ml; Sigma, St. Louis, MO, USA), and coverslips were placed on the samples with 50% glycerol in PBS.

### TUNEL staining

TUNEL staining of the sections was performed using an In Situ Cell Death Detection Kit (Roche, Germany) according to the manufacturer’s instructions. The nuclei were counterstained with DAPI.

### Western blotting

Total protein from the brain of each mouse was extracted with RIPA buffer and quantified using a Protein Reagent Assay BCA Kit (Thermo, Waltham, MA, USA). Thirty micrograms of protein from each sample was loaded and electrophoresed using 15% SDS-PAGE gels and then transferred onto a polyvinylidene difluoride (PVDF) membrane (Millipore, Billerica, MA, USA). After the membranes were blocked with 3% bovine serum albumin (BSA) at room temperature for 60 min, they were incubated with primary antibodies overnight at 4 °C. Then, the blots were incubated with secondary antibody for 2 h at room temperature (see Additional file 3: Table S2 for the antibodies used). The blots were visualized using an Infrared Imaging System (Odyssey, LI-COR, NE Lincoln, USA).

### BBB permeability assay

BBB permeability was determined by visualizing and quantifying the levels of extravasated Evans blue dye (EB) in the brain. Mice were injected intraperitoneally with 0.4 ml 2% (w/v) EB (Sigma, St. Louis, MO, USA) followed by euthanization and intracardiac perfusion with PBS 1 h later. The brains were subsequently removed and photographed. For quantification, brain tissues were weighed and homogenized in 1 ml PBS, and then 1 ml 100% trichloroacetic acid (Sangon, China) was added. The mixture was vigorously shaken for 2 min to precipitate the proteins and cooled for 30 min at 4 °C. After the mixture was centrifuged (30 min at 6000 g), the absorbance of the supernatant was measured at 620 nm using a spectrophotometer. The content of EB was quantified as nanograms of dye per gram of brain tissue using a standard curve.

### Neuro2a cells and MSC co-culture experiment

Neuro2a cells were plated in the basolateral chamber of a six-well transwell (3.0 μm, polycarbonate membrane tissue culture-treated polystyrene; Costar, Corning, NY, USA) at a density of 2 × 10^5^/well, and 1 × 10^5^ MSCs were added to the inserts and separately incubated overnight. The Neuro2a cells were then infected with JEV (multiplicity of infection (MOI) = 1). After adsorption for 1 h, the old virus suspension was removed, and fresh DMEM was added to the Neuro2a cells; subsequently, the transwell inserts containing either MSCs or DMEM alone were placed into their corresponding wells. The cells were harvested for qRT-PCR and flow cytometry after 48 h.

For mixed cultures of Neuro2a cells and MSCs, the Neuro2a cells were plated in six-well plates at a density of 2 × 10^5^/well with ten-fold diluted numbers of MSCs (ranging from 200 to 2 × 10^5^). After the cells were incubated in DMEM overnight, they were infected with JEV at an MOI = 1; 48 h later, the cells were harvested for detection of the viral load. Additionally, Neuro2a cells were plated in six-well plates at a density of 2 × 10^5^/well with MSCs (2 × 10^4^). After the cells were incubated in DMEM overnight, they were infected with JEV at a MOI = 0.1 and then harvested at 24, 48, 72, and 96 h post-infection for detection of the viral load.

### N9 cell and MSC mixed culture experiment

N9 cells were plated in six-well plates at a density of 2 × 10^5^ with or without MSCs (2 × 10^4^) and incubated overnight, after which JEV (MOI = 5) was inoculated as described above, and the cells were harvested after 48 h for flow cytometry and qRT-PCR analysis.

### Flow cytometric analysis

For annexin V/PI staining, an Annexin V-FITC Apoptosis Kit (BD Biosciences, Franklin lakes, NJ, USA) was used. The cells were washed and incubated for 15 min at room temperature with annexin V labeled with FITC and propidium iodide (PI).

Both primary cells from mouse brains and N9 cells were stained with FITC anti-mouse F4/80 and PE anti-mouse MR (CD206) for 50 min at 4 °C. After the cells were washed, they were permeabilized and stained with APC-anti-mouse iNOS for 50 min. Analysis was conducted using a BD FACSCalibur flow cytometer (BD Biosciences, San Jose, CA, USA).

### Statistical analysis

All statistical analyses were performed using GraphPad Prism version 6.01 software (La Jolla, CA, USA). Data represent the mean ± standard error of the mean (SEM). Significant differences between the experimental groups were determined using Student’s *t* test. *P* values < 0.05 were considered significant.

## Results

### MSC treatment protects mice from JEV infection-induced lethality

**Fig. 1:**
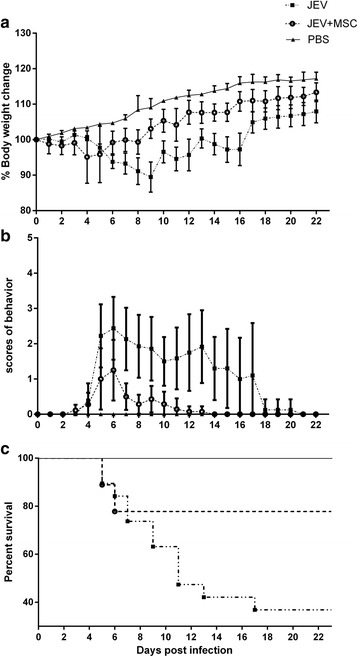
MSC treatment protects mice from JEV infection-induced lethality. Mice were given phosphate-buffered saline (*PBS*) (*n* = 7) or Japanese encephalitis virus (*JEV*) (*n* = 36) by intraperitoneal injection. Then, the mice were intravenously injected with either PBS or mesenchymal stem cells (*MSCs*) at 1 and 3 dpi via the tail vein. **a** The weight of the mice in each group was recorded between 16:30 and 17:00 daily for 22 days until all the groups were completely stable (PBS = 7, JEV = 18, JEV + MSC = 18). **b** The behavior scores were monitored and recorded twice a day according to the scoring criteria; the data are presented as the mean ± 95% confidence interval (0 = no piloerection, no restriction of movement, no body stiffening, no hind limb paralysis; 1 = piloerection, no restriction of movement, no body stiffening, no hind limb paralysis; 2 = piloerection, restriction of movement, no body stiffening, no hind limb paralysis; 3 = piloerection, restriction of movement, body stiffening, no hind limb paralysis; 4 = piloerection, restriction of movement, body stiffening, hind limb paralysis; 5 = piloerection, restriction of movement, body stiffening, hind limb paralysis, occasional tremor or death. The dead mice were scored as 5 and removed from the cohort). **c** The death and survival of the mice were recorded every day and then analyzed and presented as Kaplan–Meier survival curves

It has been shown that MSCs can relieve inflammation-induced tissue damage during the infection of viruses and bacteria through immunomodulation [32–34]. To assess the effect on the progression of JE, MSCs were transplanted at 1 and 3 dpi. In general, JEV-infected mice without MSC treatment showed clinical signs of encephalitis starting with piloerection and physical limitation followed by paresis and rigidity, and eventually progressing to severe neurological signs such as paralysis, seizure, and even death. In contrast, the MSC treatment group had a more rapid recovery in terms of weight and behavior than the JEV group (Fig. 1a and b). The survival rate was significantly improved in the MSC treatment group (77.78% survival) compared with the JEV group (36.84% survival) (Fig. 1c). These data suggested that MSC treatment alleviated the suffering and improved the prognosis in JEV-infected mice.

### MSC transplantation attenuates the inflammatory response in JEV-infected mouse brains

**Fig. 2:**
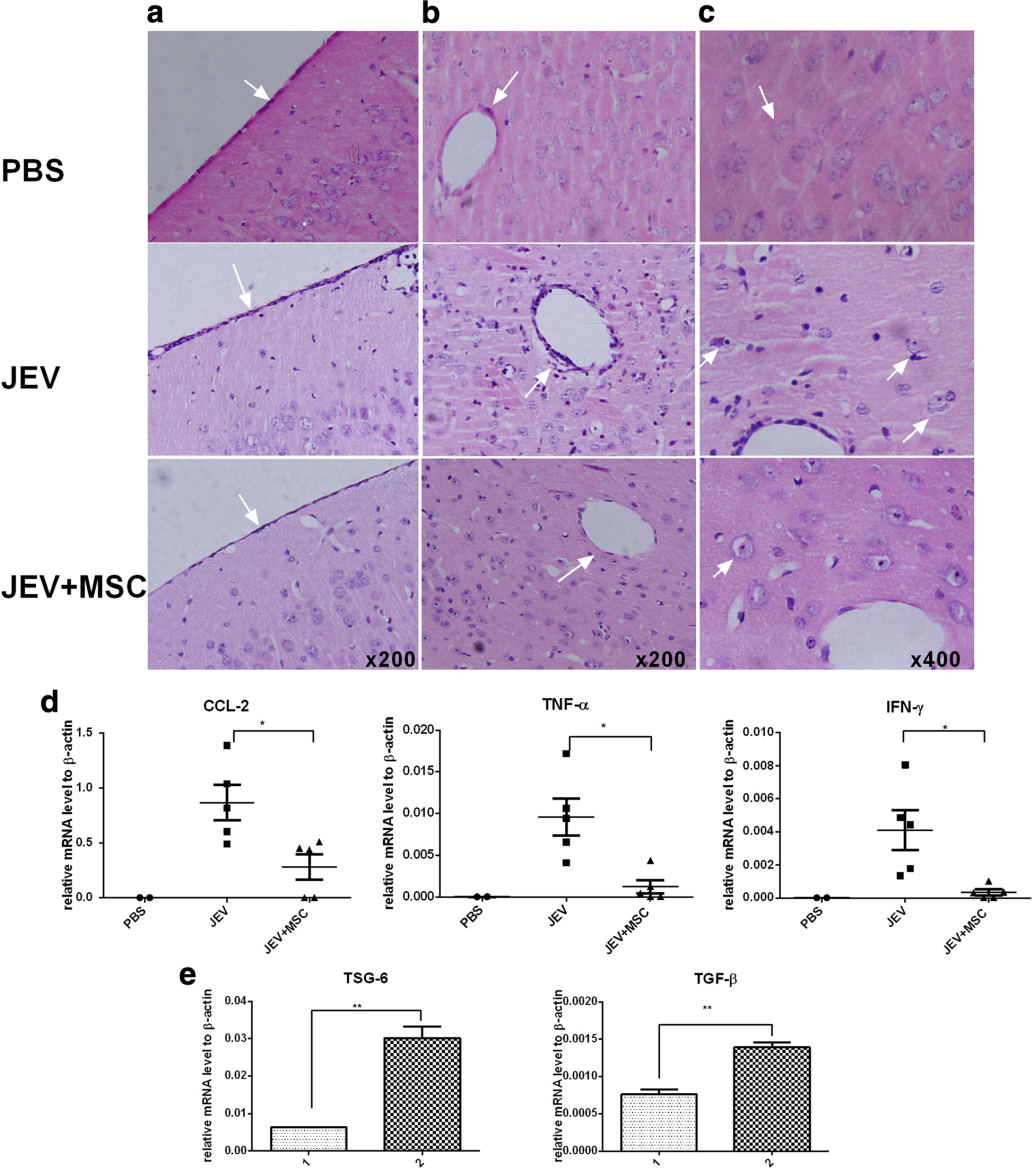
MSC transplantation attenuates the JEV-induced inflammatory response in mouse brains. Mice in each group were euthanized at 6 dpi, and the histopathological alterations and inflammatory cytokines in the brain were investigated via H&E staining and qRT-PCR, respectively. **a** Severe meningitis in Japanese encephalitis virus (*JEV*)-infected mice as indicated by *arrows*; this disease state was significantly alleviated in the mesenchymal stem cell (*MSC*) treatment group. **b** Perivascular cuffing with increased inflammatory cell infiltration was more evident in JEV-infected mice compared with the MSC treatment group. **c** Phagocytosis of neurons by inflammatory cells was apparent in JEV-infected mice (phosphate-buffered saline (*PBS*) *n*= 3, JEV *n*= 6, JEV + MSC *n*= 6). **d** Total RNA from each brain was extracted, and the inflammatory cytokine levels were tested by qRT-PCR. There was decreased expression of the inflammatory cytokines tumor necrosis factor-α (*TNF-α*), interferon-γ (*IFN-γ*), and chemokine (C-C motif) ligand 2 (*CCL-2*) in the brains from the MSC treatment group compared to JEV-infected mice (PBS *n*= 2, JEV *n*= 5, JEV + MSC *n*= 5). **e** When co-cultured with JEV-infected Neuro2a cells via transwells, the expression of TSG-6 and transforming growth factor-β (*TGF-β*) in MSCs was increased, which might contribute to the alleviated inflammation. The data represent the mean ± SEM for three independent experiments. *P < 0.05, **P < 0.01, ***P < 0.001. *1* MSCs, *2* MSCs co-cultured with JEV-infected Neuro2a cells

Neuroinflammation is a key element in the progression of JE. To observe changes in the inflammatory response between the JEV group and MSC treatment group, the histopathology and cytokine levels were detected. Histological alterations in the brain revealed severe meningitis in JEV-infected mice at 6 dpi, which was significantly alleviated in the MSC treatment group (Fig. 2a). There was more remarkable inflammatory cell infiltration in the cortexes of the JEV-infected group than in those of the MSC treatment group. Meanwhile, the level of perivascular cuffing showed a marked decrease in the MSC-treated group (Fig. 2b). There were many sieged and swallowed nerve cells in JEV-infected mice, which were rare in the MSC treatment group (Fig. 2c). The alleviated neuroinflammation in the JEV + MSC group was also observed at 10 dpi, while there was no significant difference between the JEV and JEV + MSC groups regarding meningitis and perivascular cuffing since the neuroinflammation has been resolved in survivors at 22 dpi (see Additional file 4: Figure S2). The levels of the main inflammatory cytokines and chemokines in the JEV + MSC group were generally reduced compared to the JEV group (Fig. 2d). In vitro, there was increased expression of the cytokines transforming growth factor (TGF)-β and TSG-6 in MSCs co-cultured with JEV-infected Neuro2a cells, which might play an important role in the anti-inflammatory effects (Fig. 2e). Thus MSC transplantation significantly suppressed inflammatory lesions during JE.

### MSC transplantation inhibits the activation of microglia and regulates M1-to-M2-like phenotypic switching

**Fig. 3:**
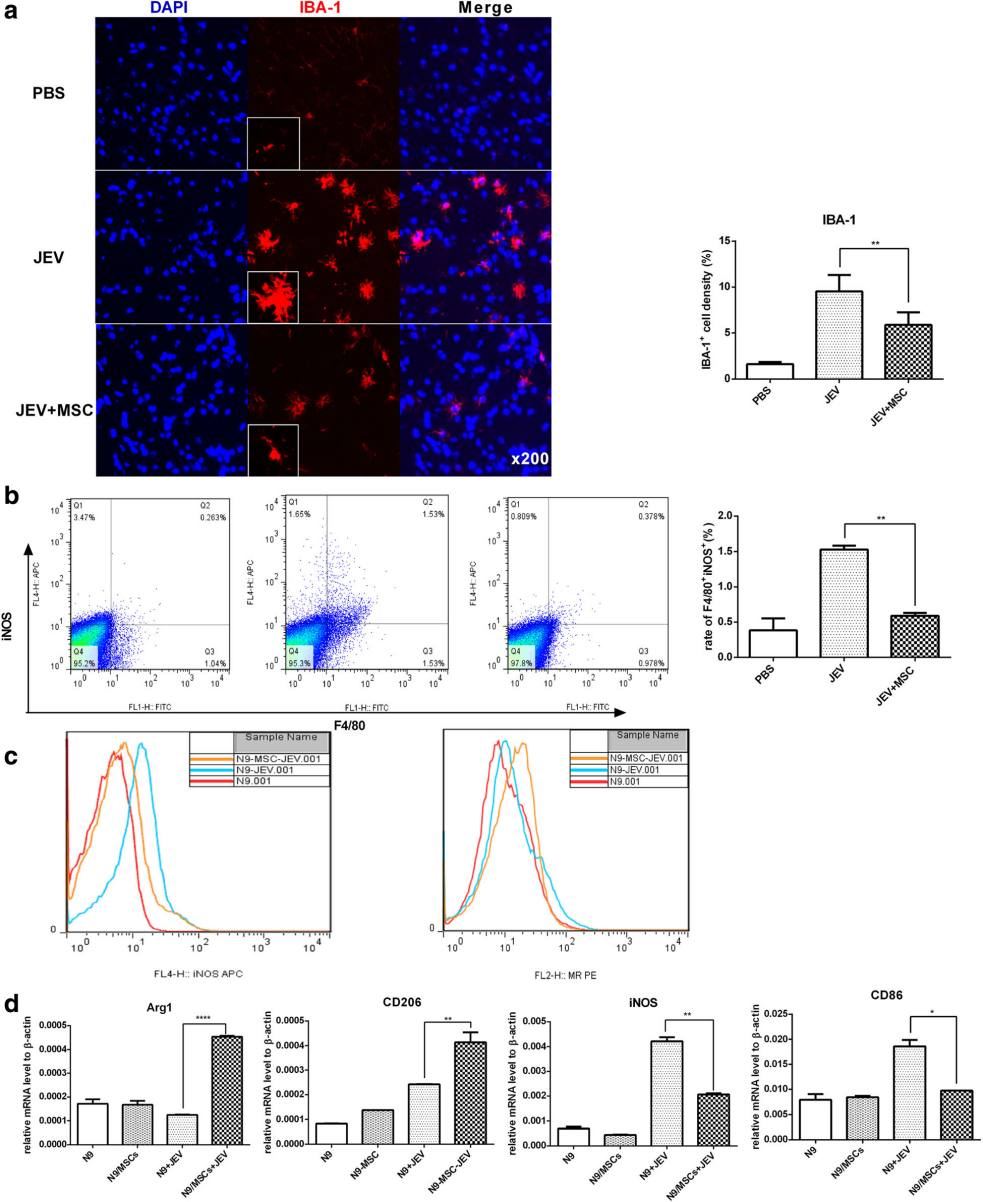
MSC transplantation inhibits the activation of microglia and regulates the M1-to-M2-like phenotypic switching. **a** The activation of microglia was detected by anti-IBA-1 antibody with the boxed areas showing a higher magnification (*left*), and the intensity of IBA^+^ cells of each group was analyzed with ImageJ (*right*) (PBS *n*= 3, JEV *n*= 6, JEV + MSCs *n*= 6; three sections per animal, five fields per section). **b** Total brain tissue was harvested, and the percentage of F4/80^+^ iNOS^+^ cells (*left*), which represents the M1 polarized macrophages in the brain, was calculated; the mean of the percentage (*right*) of M1 macrophages from each group was summarized (PBS *n*= 2, JEV *n*= 3, JEV + MSC *n*= 3). **c** In vitro cultures of the microglia line N9 with or without MSCs (10:1, respectively) were infected with JEV (MOI = 5, 48 h), and the expression levels of the M1 marker iNOS and the M2 marker MR (CD206) were analyzed by Flow Jo. **d** The expression levels of Arg1 and CD206 (markers for M2) as well as iNOS and CD86 (markers for M1) in N9 and N9-MSC cultures either with or without JEV infected with JEV (MOI = 5, 48 h) was detected by qRT-PCR. The data represent the mean ± SEM for three independent experiments. *P < 0.05, **P < 0.01, ****P < 0.0001. *Arg1* arginase-1, *IBA-1* ionized calcium binding adaptor molecule-1, *iNOS* inducible nitric oxide synthase, *JEV* Japanese encephalitis virus, *MSC* mesenchymal stem cell, *PBS* phosphate-buffered saline

Activated microglia play a central role in the pathogenesis of JE. To evaluate the effect of MSCs on microglia, ionized calcium binding adaptor molecule-1 (IBA-1), the marker of microglia activation, was stained in brain sections. Plentiful activated microglia were observed in the JEV-infected group while they were reduced in the MSC-treated group (Fig. 3a). Meanwhile, the number of inflammatory macrophages (as indicated by F4/80^+^iNOS^+^ cells) in the brain was also decreased after MSC transplantation (Fig. 3b).

It has been reported previously that different stimulation could polarize the microglia into the M1 phenotype (pro-inflammation) marked by iNOS and CD86 or the M2 phenotype (anti-inflammation) marked by Arg1 and CD206. To verify the effect of MSCs on the activation of microglia, mixed culturing of MSCs and the microglia cell line N9 was performed in vitro. The expression of iNOS and CD86 in N9 cells was induced by JEV infection, while it was decreased in the presence of MSCs. In contrast, the expression of arginase-1 (Arg1) and CD206 (which contribute to the phagocytosis of pathogens and presentation of antigen) was upregulated in N9 cells when co-cultured with MSCs (Fig. 3c and d). Thus, MSC transplantation inhibited JEV-induced overactivation of microglia as well as regulated the M1-to-M2-like phenotypic switching.

### MSCs reduce neuronal death in vivo and in vitro

**Fig. 4:**
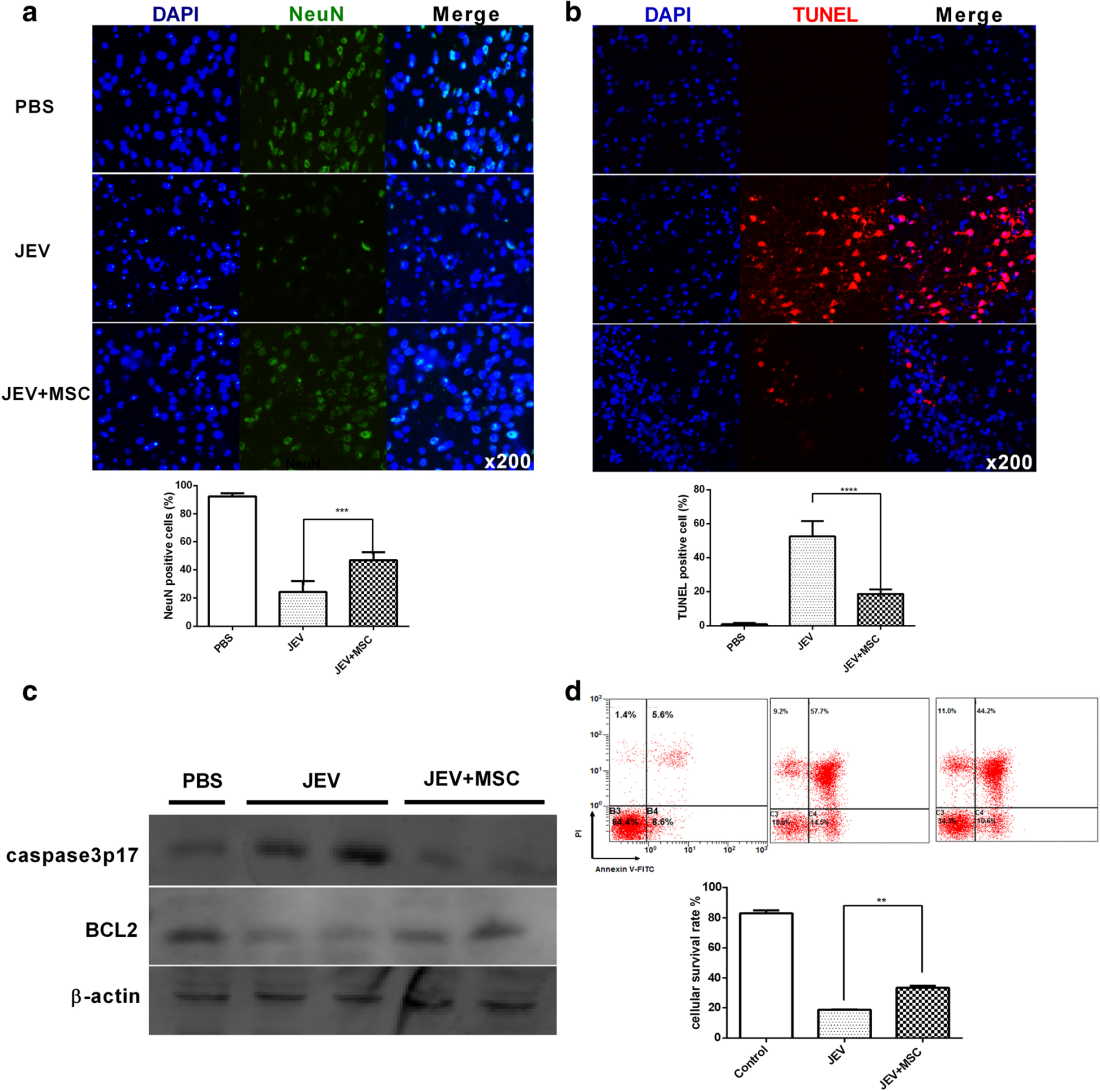
MSCs reduce neuronal death in vivo and in vitro. Mice undergoing different treatments were euthanized at 6 dpi. **a** The survival rate of neurons was detected with anti-NeuN antibody staining. **b** The TUNEL assay was performed to detect neuronal apoptosis, and the sections from each group were analyzed with ImageJ. The positively stained cells were counted (PBS *n*= 3, JEV *n* ﻿= 6, JEV + MSC *n*= 6; three sections per mouse, five fields per section). **c** The total protein of whole brain tissue was extracted, and the expression levels of the active caspase-3 subunit P17 (caspase-3 p17) and BCL2 in the brains of each group were detected by Western blot (PBS *n*= 1, JEV *n*= 2, JEV + MSC *n*= 2). **d** Neuro2a cells were cultured via transwells either with or without MSCs and were infected with JEV at MOI = 1. After 48 h, annexin V/PI staining was performed, and the mean of the survival rate was summarized from three independent experiments. **P < 0.01, ***P < 0.001, ****P < 0.0001. *BCL2* B-cell lymphoma-2 protein, *JEV* Japanese encephalitis virus, *MSC* mesenchymal stem cell, *NeuN* neuron-specific nuclear protein, *PBS* phosphate-buffered saline, *PI* propidium iodide

Massive neuronal death leads to progression and deterioration of JE. To explore whether MSCs could improve the neuronal viability, neuron-specific nuclear protein (NeuN; a marker of neurons)-positive cells were counted in brain sections from the different groups. There were more surviving neurons in the MSC-treated mice (Fig. 4a). According to the TUNEL assay (Fig. 4b) and Western blot (Fig. 4c), MSC treatment effectively decreased neuronal apoptosis. There was a decreased level of active caspase-3 subunit P17 and increased expression of B-cell lymphoma-2 protein (BCL2; an anti-apoptotic protein) in MSC-treated mouse brains compared with the JEV group. In vitro, the direct role of MSCs on neurons was evaluated through transwell co-culture. More Neuro2a cells survived when co-cultured with MSCs than did solo cultures (Fig. 4d). These data indicated that MSC treatment reduced neuronal loss.

### MSC transplantation alleviates virus-induced BBB destruction and improves the expression of ZO-1 in the mouse brain

**Fig. 5:**
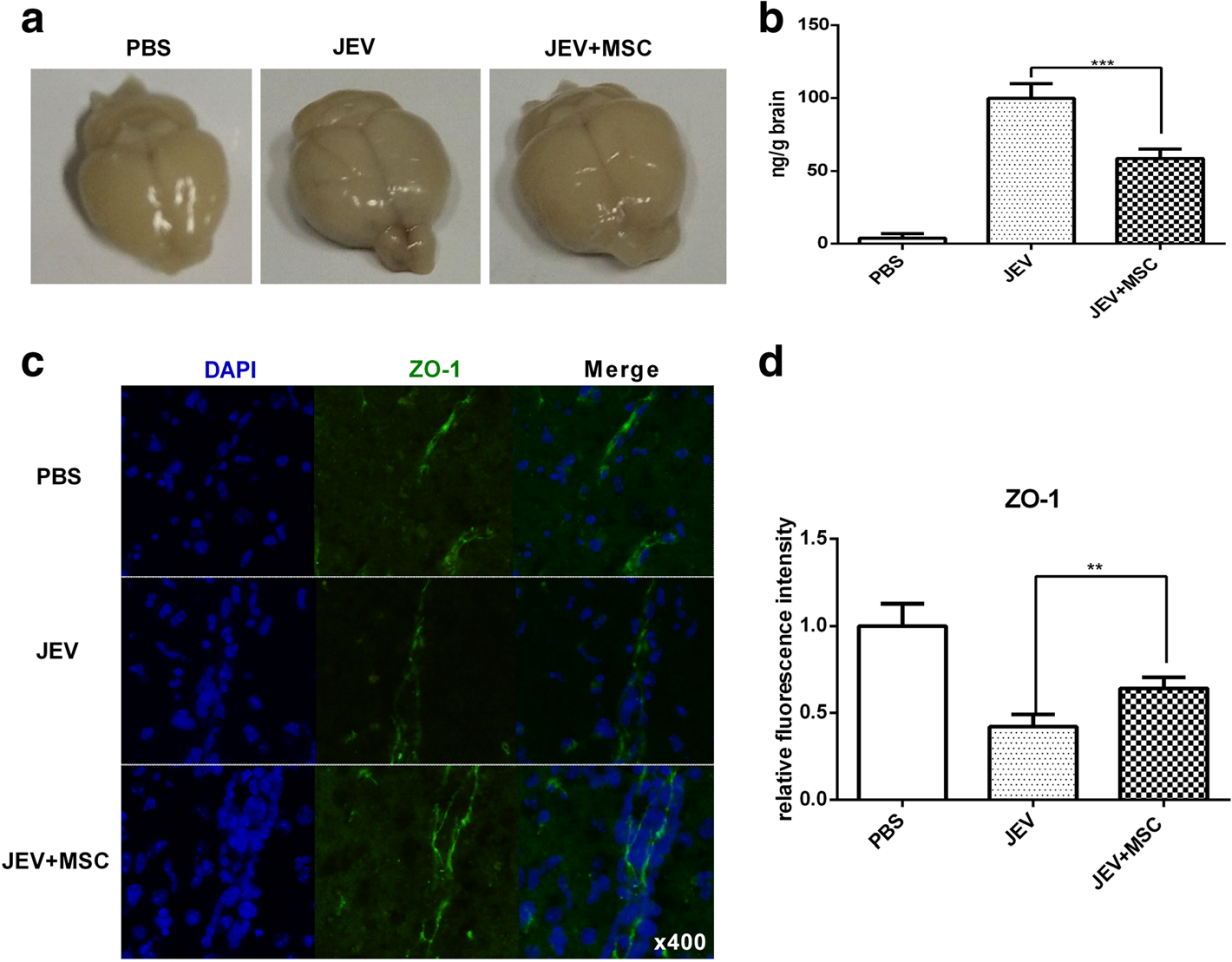
MSC transplantation alleviates virus-induced disintegration of the BBB and improves the expression of ZO-1 in the mouse brain. Mice from each group were euthanized to detect the integrity of the BBB at 6 dpi. **a** BBB permeability was evaluated by the uptake of Evans blue dye assay (EB), and the brains were removed and photographed. These pictures show extravasated EB staining of the whole brain at 6 dpi. **b** The absorbance of EB in the brain was measured at 620 nm using a spectrophotometer and recorded as nanograms of dye per gram of brain tissue (PBS *n*= 2, JEV *n*= 3, JEV + MSC *n*= 3). **c** The expression of the tight junction protein zonula occludens-1 (*ZO-1*) in the brain sections was assessed by immunofluorescence. **d** The fluorescence intensity of ZO-1 staining was analyzed using ImageJ and showed that MSC treatment increased the level of ZO-1 compared with the JEV-infected group (PBS *n*= 3, JEV *n*= 6, JEV + MSC *n*= 6; three sections per mouse, five fields per section). **P < 0.01, ***P < 0.001. *JEV* Japanese encephalitis virus, *MSC* mesenchymal stem cell, *PBS* phosphate-buffered saline

It has been established that MSCs could regulate epithelial cells and stabilize the integrity of the BBB in many CNS diseases through a paracrine effect [32]. To determine the effect of MSC treatment on BBB integrity during JEV infection, an EB assay was performed. In general, there was no significant difference in the appearance of color between control and JEV + MSC groups. However, the brains from JEV-infected mice were slightly tinged blue (Fig. 5a). Based on the amount of EB retained in the brains, mice in the JEV + MSC group showed reduced permeability of the BBB compared with the JEV-infected group (Fig. 5b). Furthermore, there was higher expression of zonula occludens-1 (ZO-1) in the JEV + MSC group than the JEV-infected group (Fig. 5c and d). Therefore, MSC treatment could improve the expression of ZO-1 and reduce the JEV-induced breakdown of the BBB.

### MSCs prevent JEV propagation via interferon expression

**Fig. 6:**
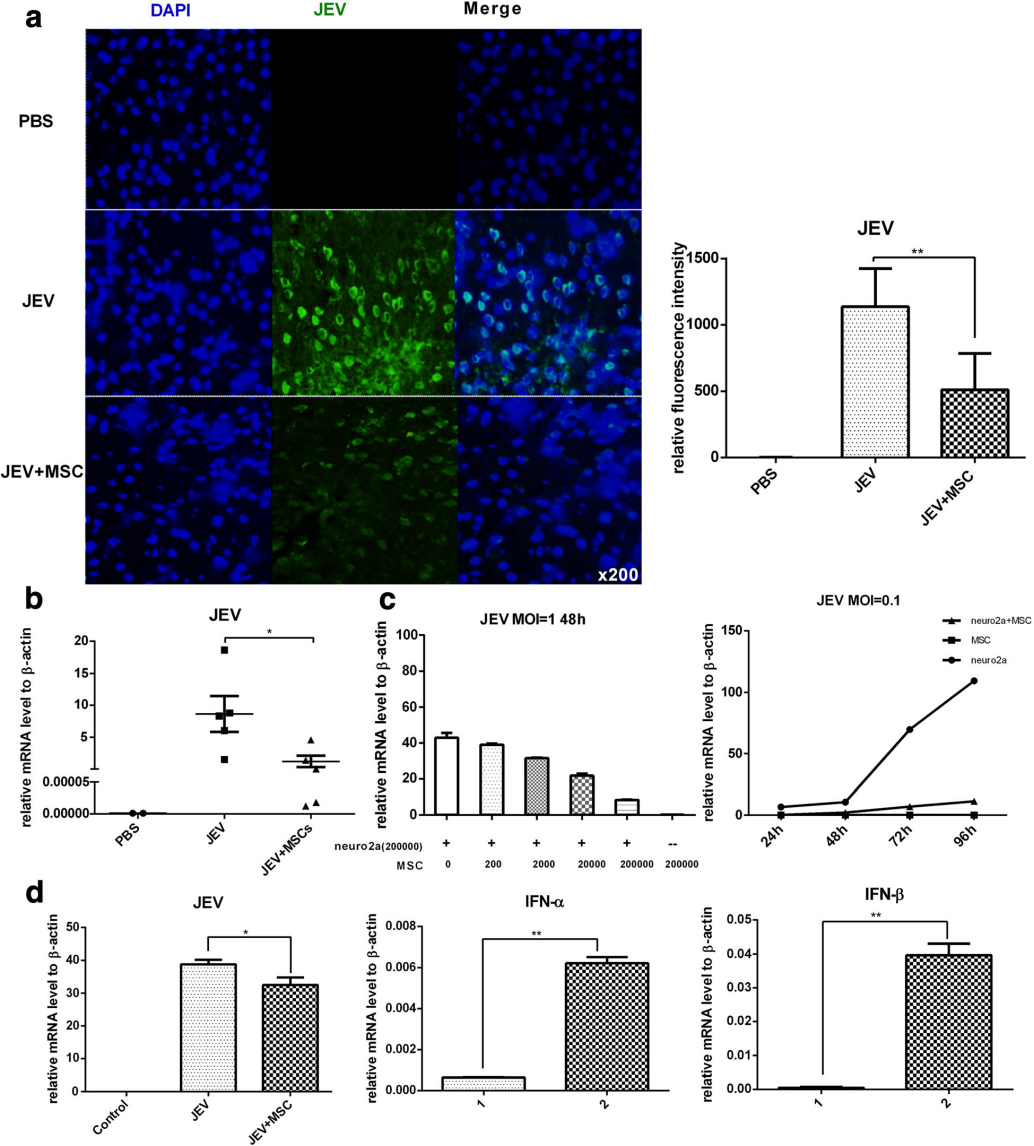
MSCs prevent JEV propagation via interferon expression. **a** The viral load of JEV in the brain was assessed by detecting E protein through immunofluorescence, and the fluorescence intensity was analyzed using ImageJ (PBS *n*= 3, JEV *n*= 6, JEV + MSC *n*= 6; three sections per mouse, five fields per section). The staining showed that MSC treatment decreased the viral load in mice brains at 6 dpi. **b** Total RNA of the whole brain from each mouse was extracted, and the viral load of JEV was assessed by qRT-PCR (PBS *n*= 2, JEV *n*= 5, JEV + MSC *n*= 5) at 6 dpi. **c** For the in vitro experiments, Neuro2a cells were co-cultured with different numbers of MSCs and infected with JEV at multiplicity of infection (*MOI*) = 1, and the viral titer was detected 48 h later by qRT-PCR (*left panel*). Neuro2a cells and MSCs were co-cultured at a ratio of 1:1, and JEV was added at MOI = 0.1. The cells were then harvested at 24, 48, 72, and 96 h after infection, and the viral titer was determined by qRT-PCR (*right panel*). **d** Neuro2a cells were co-cultured with MSCs in a noncontact transwell system at a ratio of 10:1, and Neuro2a cells were infected with JEV at MOI = 1. The Neuro2a cells and MSCs were harvested 48 h later, and the viral copy number in Neuro2a cells and the expression of interferon (*IFN*)-β and IFN-α in MSCs was detected by qRT-PCR. The data represent the mean ± SEM for three independent experiments. *P < 0.05, **P < 0.01. *1* MSCs, *2* MSCs co-cultured with JEV-infected Neuro2a cells, *JEV* Japanese encephalitis virus, *MSC* mesenchymal stem cell, *PBS* phosphate-buffered saline

Although MSCs inhibited the neuroinflammation induced by JEV infection, it was unknown whether the alleviated inflammation led to uncontrolled amplification of the virus. Thus, we tested the titer of JEV in the brains at 6 dpi from each group by immunofluorescence (Fig. 6a) and qRT-PCR (Fig. 6b). The viral load was generally decreased in the JEV + MSC group compared with the JEV group.

To verify the role of MSCs on JEV proliferation, a series of mixed cultures of Neuro2a cells and MSCs were conducted. The viral load was decreased as the number of co-cultured MSCs increased (Fig. 6c, left). Meanwhile, there was significantly lower viral copy and slower proliferation in Neuro2a cells when co-cultured with MSCs compared with the control group (Fig. 6c, right). Besides, through transwell co-culture system, the viral copy number in the Neuro2a cells was reduced accompanied with increased expression of IFN-β and IFN-α in MSCs (Fig. 6d). Therefore, these data indicated that MSCs could inhibit the propagation of JEV in Neuro2a cells.

## Discussion

Recently, there have been an increasing number of studies on the therapeutic effect of MSCs on CNS diseases and virus infection. It was reported that MSCs promoted neurological recovery via regulating neuroinflammation and secreting neurotrophic factors. In addition, exogenous MSC transplantation has been demonstrated to reduce influenza virus-induced acute lung injury [32, 34]. In our study, we found that MSC transplantation improved the survival rate and alleviated neurological symptoms in a JEV-infected mouse model. These findings are the first evidence that MSCs could serve as a potential adjunctive therapy for JE.

There are many ways to construct a JEV infection mouse model, such as intracerebral injection, intravenous injection, and intraperitoneal injection. Based on feasibility and noninvasion, we adopted intraperitoneal injection for our study. In this study, mice older (8–10 weeks of age) than those previously reported in a JE mouse model (4–6 weeks of age) were used, and the dose of inoculated JEV was slightly higher than the median lethal dose (LD_50_). In our preliminary experiments, there was no obvious difference in the neurological symptoms and histopathology between the younger and older mouse model. JEV-infected mice generally began to exhibit typical symptoms at 3 dpi. There was an increased risk of pulmonary embolism when the number of intravenously transplanted cells was more than 5 × 10^5^, and thus two intravenous injections of 5 × 10^5^ cells/mouse were conducted at 1 and 3 dpi. In this study, we focused on the effect of MSCs on the acute neuroinflammation; thus, brains from each group were collected at 6 dpi.

The overproduction of inflammatory cytokines is the main cause of neuropathology during JE. Thus, inhibiting the inflammation during the progression of JE has been shown to reduce neuronal death and promote neurogenesis [35]. An appropriate immune and inflammatory response during the early phase of infection is critical for clearance of the virus and regeneration of the nervous system [36]. However, the overproduction of IFN-γ and TNF-α accelerated the neuroinflammation, and neutralization of these inflammatory cytokines could reduce the neuronal damage during JEV infection [37, 38]. Thus, it is important to achieve a balance between immune and inflammatory responses for the treatment of JE. MSCs are characterized by immunoregulation and have shown great potential in the treatment of immune deficiencies and infectious diseases [25, 39]. In this experiment, MSC-treated mice showed decreased level of IFN-γ and TNF-α at 6 dpi. Although the effect of these decreased cytokines in the course of JE is uncertain, it was well demonstrated that the neuroinflammation was alleviated in the MSC-treated mice according to tissue pathological changes. The dynamic regulation of MSCs during JE, which might be involved in the increased immune defenses to speed up the clearance of virus in the early stage of infection, is worth further exploration.

Previous studies have shown that breakdown of the BBB can exacerbate the progression of JE [13]. It has been demonstrated that MSCs can improve the barrier function of epithelial cells through the production of the growth factors Ang1 and KGF, as well as regulating the integrity of the BBB through the expression of TIMP3 (tissue inhibitor of matrix metalloproteinase-3) [32]. Our results indicated that the BBB permeability was ameliorated in MSC transplanted mice compared to the PBS-treated group. One possible explanation is that MSCs could inhibit the excessive production of inflammation cytokines and the overactivation of glia, both of which can lead to the degradation of ZO-1 [13] and the subsequent destruction of the BBB. However, the direct effect of MSCs on the BBB during JEV infection is still being explored.

It is well known that activation of microglia can trigger a secondary pathway of damage and influence the outcome of JEV progression [11, 40]. Our data indicated that MSCs could inhibit the activation of microglia. Meanwhile, when co-cultured with MSCs, JEV-challenged N9 microglia cells showed increased expression of Arg1 and CD206, the markers of the M2 phenotype. These data are consistent with a previous study that MSCs reprogrammed microglia into a M2-like phenotype to inhibit the overproduction of inflammatory mediators and promote the regeneration of tissue [41].

After JEV infection either in vivo or in vitro, there is a significant production of TNF-α. It has been demonstrated that MSCs can secrete TSG-6 (TNF-α stimulated gene/protein 6) upon the induction of TNF-α to modulate inflammatory responses, inhibit the destruction of the BBB, and ameliorate tissue injury [42, 43]. In vitro, we found that upon co-culturing with JEV-infected Neuro2a cells, MSCs showed increased expression of TSG-6 and TGF-β, and the Neuro2a cells exhibited decreased inflammatory cytokine expression and increased survival. The role and mechanism of TSG-6 from MSCs in the alleviation of neuroinflammation during JE are still in research.

In addition to the regulation of inflammation, the immune defense function of MSCs has also been demonstrated in many bacteria- and virus-induced injuries [32, 33, 44]. To our knowledge, there are few reports about the antiviral role of MSCs. Interestingly, we found statistically decreased viral load in MSC-treated mice. In addition, the JEV titer in Neuro2a cells was decreased when co-cultured with MSCs. One reason for this phenomenon is that the immunomodulation of MSCs can improve the innate and adaptive immune responses and contribute to the elimination of virus. Moreover, in this study, MSCs co-cultured with JEV-infected Neuro2a cells showed significantly increased expression of IFN-β and IFN-α compared with control cells. It is well known that type I interferons play a key role in the defense against JEV [45]. Furthermore, a series of changes in genes related to antiviral activity in MSCs were detected after either JEV stimulation or co-culturing with JEV-infected Neuro2a cells through gene expression profiling in our study (data unpublished). It has been reported that MSCs can exert antibacterial effects by secretion of beta-defensin-2 via Toll-like receptor 4 (TLR4) [33]. Whether MSCs have a direct antiviral effect and the exact mechanism is currently being explored.

Recently, engineered MSCs have also been developed to exert more effective antiviral activities. As previously reported, MSCs with a transgene encoding humanized Venezuelan equine encephalitis virus (VEEV) neutralizing antibody (anti-VEEV) had a more efficacious protective effect in nude mice infected with VEEV [46]. However, the protection of sham MSCs transplanted via intramuscular injection was poor in the VEEV infection model [46]. In our study, we found that MSC transplantation via intravenous injection increased the survival and reduced the weight loss of JEV-infected mice. This divergence between the VEEV and JEV results might due to the different pathogenic mechanisms, mouse models used, and transplantation means applied.

In this study, we focused on the effects of MSCs on the morbidity and acute neuroinflammation during JEV infection and harvested mouse brains at 6 dpi. Whether MSC transplantation improves neurological function and prognosis during the late stage of encephalitis is also being explored by our group.

## Conclusion

Taken together, this study demonstrated that MSCs exerted beneficial effects against JE in the mouse model. It is the first evidence that intravenous MSCs could serve as a potential adjunctive therapy for JE. Whether this treatment modality can be applied to other types of viral encephalitis warrants further investigation.

## Additional files

Additional file 1: Figure S1. Characterization of mouse MSCs. (A) The typical spindle-shape morphology of MSCs. (B,C) Analysis of the surface makers on MSCs by flow cytometry. The positive markers CD44 and Sca-1 are >95% (B) and the negative markers I-A/I-E, CD34, and CD45 are <5% (C). (D) Differentiation of MSCs into adipocytes, osteocytes and chondrocytes. (JPG 4926 kb)
Additional file 2: Table S1. Primer sequences used in this study. (DOCX 15 kb)
Additional file 3: Table S2. Primary antibodies used in this study. (DOCX 14 kb)
Additional file 4: Figure S2. At 10 and 22 dpi, mouse brains were collected as described and the standard H&E staining protocol was followed. (A) Severe meningitis in JEV-infected mice as indicated by arrows; this disease state was significantly alleviated in the MSC treatment group at 10 dpi. (B) Perivascular cuffing with increased inflammatory cell infiltration was more evident in JEV-infected mice compared with the MSC treatment group at 10 dpi. (C) Phagocytosis of neurons by inflammatory cells was apparent in JEV-infected mice at 10 dpi, while there was no significant difference between the JEV and JEV + MSC groups regarding meningitis, perivascular cuffing, and neuronal damage since the neuroinflammation had been resolved at 22 dpi. (JPG 4626 kb)

## Abbreviations

Arg1: Arginase-1
BBB: Blood-brain barrier
BCL-2: B-cell lymphoma-2 protein
BSA: Bovine serum albumin
CCL-2: Chemokine (C-C motif) ligand 2
CNS: Central nervous system
DAPI: 4′,6-Diamidino-2-phenylindole
dpi: Days post-infection
EB: Evans blue dye
FBS: Fetal bovine serum
FMMU: Fourth Military Medical University
H&E: Hematoxylin and eosin
IBA-1: Ionized calcium binding adaptor molecule-1
IFN: Interferon
IHC: Immunohistochemical
iNOS: Inducible nitric oxide synthase
JE: Japanese encephalitis
JEV: Japanese encephalitis virus
L-DMEM: Low-glucose Dulbecco’s modified Eagle’s medium
MOI: Multiplicity of infection
MSC: Mesenchymal stem cell
NeuN: Neuron-specific nuclear protein
PBS: Phosphate-buffered saline
PFA: Paraformaldehyde
PI: Propidium iodide
qRT-PCR: Quantitative real-time polymerase chain reaction
SDS-PAGE: Sodium dodecyl sulfate polyacrylamide gel electrophoresis
SEM: Standard error of the mean
TGF: Transforming growth factor
TNF: Tumor necrosis factor
TUNEL: Terminal-deoxynucleotidyl transferase-mediated nick-end labelling
VEEV: Venezuelan equine encephalitis virus
ZO-1: Zonula occludens-1
