# Supplementary figures and images for: Mesenchymal stem cells alleviate Japanese encephalitis virus-induced neuroinflammation and mortality
Source: Stem Cell Res Ther. 2017 Feb 16;8:38. doi: 10.1186/s13287-017-0486-5 (PMC5314473; doi:10.1186/s13287-017-0486-5)

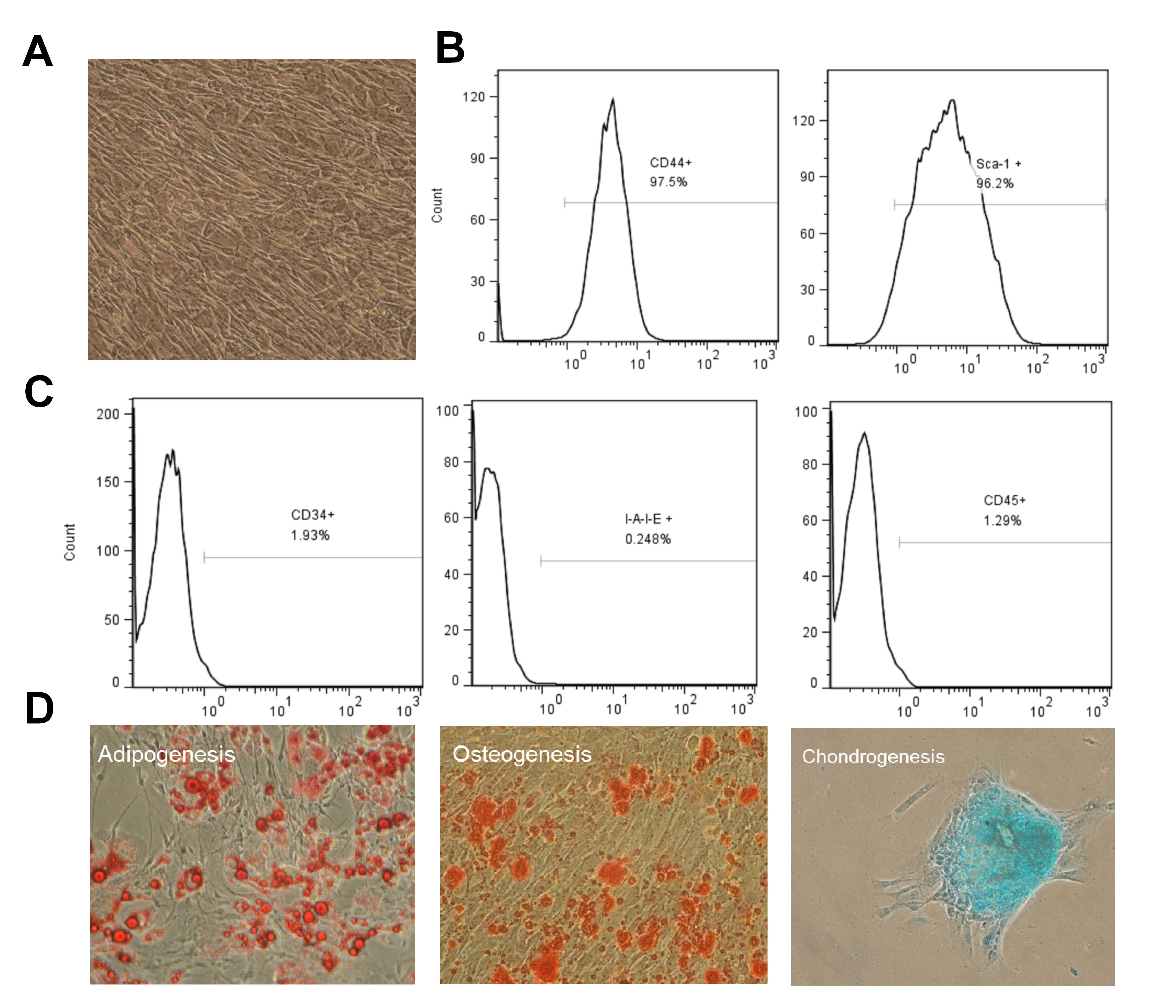

Supplement: Additional file 1: Figure S1. — Characterization of mouse MSCs. (A) The typical spindle-shape morphology of MSCs. (B,C) Analysis of the surface makers on MSCs by flow cytometry. The positive markers CD44 and Sca-1 are >95% (B) and the negative markers I-A/I-E, CD34, and CD45 are <5% (C). (D) Differentiation of MSCs into adipocytes, osteocytes and chondrocytes. (JPG 4926 kb) [file 13287_2017_486_MOESM1_ESM.jpg]

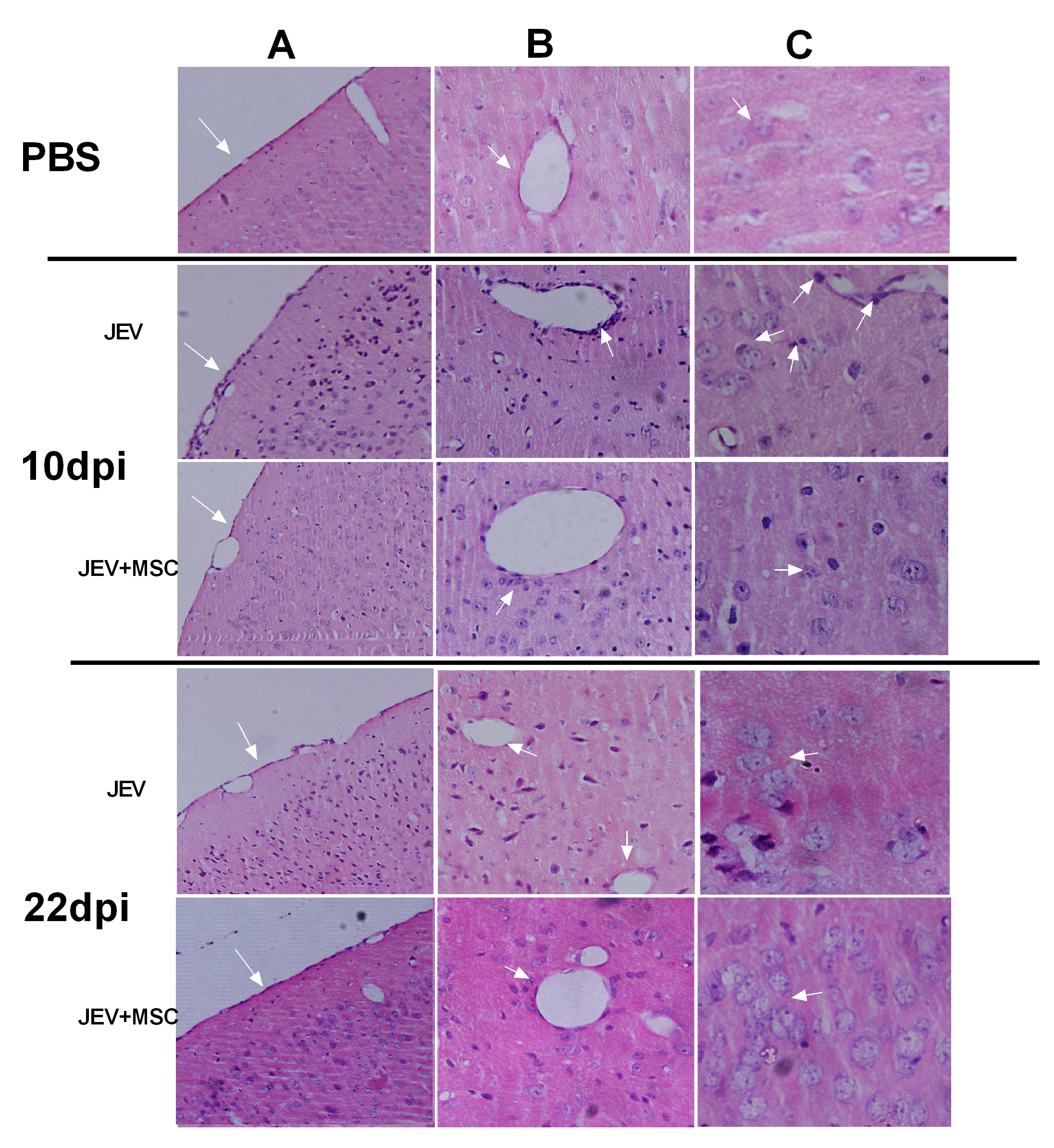

Supplement: Additional file 4: Figure S2. — At 10 and 22 dpi, mouse brains were collected as described and the standard H&E staining protocol was followed. (A) Severe meningitis in JEV-infected mice as indicated by arrows; this disease state was significantly alleviated in the MSC treatment group at 10 dpi. (B) Perivascular cuffing with increased inflammatory cell infiltration was more evident in JEV-infected mice compared with the MSC treatment group at 10 dpi. (C) Phagocytosis of neurons by inflammatory cells was apparent in JEV-infected mice at 10 dpi, while there was no significant difference between the JEV and JEV + MSC groups regarding meningitis, perivascular cuffing, and neuronal damage since the neuroinflammation had been resolved at 22 dpi. (JPG 4626 kb) [file 13287_2017_486_MOESM4_ESM.jpg]
