# Supplementary material for: Mesenchymal stem cells alleviate Japanese encephalitis virus-induced neuroinflammation and mortality
Source: Stem Cell Res Ther. 2017 Feb 16;8:38. doi: 10.1186/s13287-017-0486-5 (PMC5314473; doi:10.1186/s13287-017-0486-5)
Supplement: Additional file 2: Table S1. — Primer sequences used in this study. (DOCX 15 kb) [file 13287_2017_486_MOESM2_ESM.docx]

| JEV | F: AGACAAGCAGATCAACCACCATT R: CCCTCCAATAGAGCCAAAGTCC |
| --- | --- |
| TNF-α | F: CTG AAC TTC GGG GTG ATC GGT R: ACG TGG GCT ACA GGC TTG TCA |
| IFN-γ | F: GGCCATCAGCAACATAAGCGT R: TGGGTTGTTGACCTCAAACTTGGC |
| IFN-α | F:TCC TGA ACC TCT TCA CAT CAA A R: ACA GGC TTG CAG GTC ATT GAG |
| IFN-β | F: CTCCACCACAGCCCTCTC R: CATCTTCTCCGTCATCTCCATAG |
| TGF-β | F: ATCCTCAAGTTGCACCCTTATCT R: AAAGAGCCTTCGGTGGATTGC |
| TSG-6 | F: GGCTGGCAGATACAAGCTCA R: TCAAATTCACATACGGCCTTGG |
| iNOS | F: CCCTTCAATGGTTGGTACATGG R: ACATTGATCTCCGTGACAGCC |
| CD86 | F: TTGTGTGTGTTCTGGAAACGGAG R: AACTTAGAGGCTGTTGCTGGG |
| CD206 | F: TCTTTGCCTTTCCCAGTCTCC R: TGACACCCAGCGGAATTTC |
| Arg1 | F: GAACACGGCAGTGGCTTTAAC R: TGCTTAGCTCTGTCTGCTTTGC |
| CCL-2 | F: CAA GAA GGA ATG GGT CCA GA R: GCT GAA GAC CTT AGG GCA GA |
| β-Actin | F: TGACGGGGTCACCCACACTG R: AAGCTGTAGCCGCGCTCGGT |

Table S1. The primer sequences used in this study
