# Supplementary material for: Mesenchymal stem cells alleviate Japanese encephalitis virus-induced neuroinflammation and mortality
Source: Stem Cell Res Ther. 2017 Feb 16;8:38. doi: 10.1186/s13287-017-0486-5 (PMC5314473; doi:10.1186/s13287-017-0486-5)
Supplement: Additional file 3: Table S2. — Primary antibodies used in this study. (DOCX 14 kb) [file 13287_2017_486_MOESM3_ESM.docx]

| **Antibodies** | **Hosts** | **Dilutions** | **Sources** |
| --- | --- | --- | --- |
| Anti-IBA-1 | Goat | IHC 1:500 | Abcam England |
| Anti-NeuN | Rabbit | IHC 1:900 | Abcam England |
| Anti-JEV | Mouse | IHC 1:100 | China |
| Anti-ZO-1 | Rabbit | IHC 1:100 | Proteintech China |
| Anti-caspase3p17 | Rabbit | WB 1:1000 | Millipore USA Massachusetts |
| Anti-BCL-2 | Rabbit | WB 1:500 | Proteintech China |
| Anti-β-actin | Mouse | WB 1:1000 | Proteintech China |
| PE-anti-CD44 | Rat | FCM 1:100 | BD USA New Jersey |
| FITC-anti-Sca-1 | Rat | FCM 1:100 | BD USA New Jersey |
| FITC-anti-CD45 | Rat | FCM 1:100 | BD USA New Jersey |
| PE-anti-I-A/I-E | Rat | FCM 1:100 | BD USA New Jersey |
| FITC-anti-CD34 | Rat | FCM 1:100 | BD USA New Jersey |
| FITE-anti-F4/80 | Rat | FCM 1:100 | Biolegend USA California |
| PE-anti-MR | Rat | FCM 1:100 | Biolegend USA California |
| APC-anti-iNOS | Rat | FCM 1:100 | Biolegend USA California |
| AF-488-anti-rabbit IgG | donkey | IHC1:200 | Proteintech China |
| Cy3-anti-goat IgG | donkey | IHC1:200 | Proteintech China |
| AF-488-anti-mouse IgG | rabbit | IHC1:200 | Proteintech China |
| DyLight 800-anti-Mouse IgG | goat | WB 1:10000 | BD USA New Jersey |
| DyLight 680-anti-rabbit IgG | goat | WB 1:10000 | BD USA New Jersey |

Table S2. The antibodies used in this study.

For IHC, the primary antibodies were diluted with 0.01 M phosphate buffered saline (PBS) containing 0.1 % Triton X-100 and 1 % bovine serum albumin (BSA). For WB, the primary antibodies were diluted with 0.01 M PBS containing 3 % BSA. For flow cytometry, antibodies were diluted with 0.01 M PBS containing 2% FBS. The secondary antibodies were diluted with 0.01M PBS.
